# The 2019 Rio Grande birth cohort: profile of a Brazilian 5-year study on mental health conditions

**DOI:** 10.4178/epih.e2025039

**Published:** 2025-07-21

**Authors:** Rafaela Costa Martins, Francine dos Santos Costa, Cauane Blumenberg, Thais Martins-Silva, Romina Buffarini, Juraci Almeida Cesar, Christian Loret de Mola

**Affiliations:** 1 Grupo de Pesquisa e Inovação em Saúde (GPIS), Federal University of Rio Grande (FURG), Rio Grande, Brazil; 2 Human Development and Violence Research Centre (DOVE), Federal University of Pelotas (UFPel), Pelotas, Brazil; 3 Causale Consultoria, Pelotas, Brazil; 4 Universidade Federal de Pelotas, Centro de Equidade, Pelotas, Brazil; 5 Post-Graduate Program in Health Sciences, Federal University of Rio Grande (FURG), Rio Grande, Brazil; 6 Post-Graduate Program in Public Health, Federal University of Rio Grande (FURG), Rio Grande, Brazil; 7 CHANGE Research Working Group, Universidad Científica del Sur, Lima, Peru

**Keywords:** Birth cohort, Mental health, Maternal health, Child health, Life change events

## Abstract

We established the 2019 Rio Grande birth cohort to investigate the life course epidemiology of mental health and its intergenerational transmission. In 2019, we systematically identified all hospital births in the city of Rio Grande, located in Southern Brazil. Mothers who delivered a singleton liveborn child were administered a standardized, face-to-face questionnaire. In 2020, we conducted 2 follow-up assessments (WebCOVID-19 1 and 2), a third in 2021-22 (WebCOVID-19-3), and a fourth in 2023-24 (WebPOST-COVID). Across these follow-ups, we collected data on socio-demographic, environmental, and behavioral factors pertaining to both mother and child, as well as maternal mental health. Child mental health and development were specifically evaluated during the fourth follow-up. At baseline, 2,051 mothers were interviewed. Response rates for the online follow-ups were 54.1%, 51.1%, 48.7%, and 34.6%, respectively. In WebCOVID-19-3, the highest prevalence rates for depression (34.7%) and anxiety (33.1%) were observed. This cohort provides novel insights into maternal mental health, child development, and post-coronavirus disease 2019 behaviors, emphasizing culturally specific risk factors. Our findings are based on both published and ongoing studies. Data may be requested upon reasonable request.

## INTRODUCTION

Nearly 800 million people worldwide live with mental health disorders, and depressive disorders are among the leading causes of years lived with disability (YLD) and disability-adjusted life years (DALYs) [1]. Despite decades of research, reducing the risk of mental health disorders remains challenging, especially in low-income and middle-income countries (LMICs). In LMICs, trends in mental health burden have shown variable patterns over the past 30 years; however, in Brazil, DALYs have steadily increased by approximately 50% [2]. The burden of anxiety, as measured by DALYs and YLDs, is among the highest globally in Brazil, and for females, the burden is nearly double that observed in males [1,2]. More than 8 million Brazilians are estimated to suffer from depression, with 60% being female [1-3].

Inequality and social adversity, particularly in Brazil, contribute to the high prevalence of mental health issues, especially among females [1,4]. Although Brazil has a universal healthcare system, the treatment gap remains significant, as is the case in most LMICs [5].

The coronavirus disease 2019 (COVID-19) pandemic in 2019 led to a global increase in mental health problems [6], higlighting disparities in LMICs. The long-term effects of this catastrophic event on the mental health of mothers and their children remain unknown. Maternal mental health profoundly impacts parenting and child development, with depression linked to behavioral problems and a heightened risk of depression in children [7]. Intergenerational transmission is shaped by genetics, shared environment, and direct maternal behavior, with the latter being the only modifiable factor to improve outcomes for both mothers and children [8-10].

Understanding how maternal mental health influences child development is of paramount importance given the rising mental health burden. Mental health conditions result from a complex interplay of factors across the life course, including the intergenerational transmission of risk factors [10,11]. Maternal mental health significantly affects parenting, child development, and the child’s mental health [9].

Few studies have prospectively and longitudinally examined the impact of maternal characteristics and early-life adversity on developmental health in LMICs [12,13]. This is critical, as health patterns may differ between LMICs and high-income countries, where most evidence originates. Factors such as social inequalities, parenting behaviors, and sustained exposure to adverse environments may influence mental health outcomes [14]. More research is thus needed to examine these factors across diverse contexts to draw accurate conclusions.

Current research has clarified “why” children of parents with mental health disorders are more likely to develop similar problems, but the “how” still requires further investigation. Despite strong evidence that early childhood experiences affect later life stages and that maternal health impacts child health [15-17], more research is needed to understand how maternal context and psychological disorders shape mother-infant relationships, child development, and overall health in LMICs.

Other regional birth cohorts, such as the Pelotas cohorts, have made significant contributions to life course epidemiology on health exposures and outcomes [12,18]. However, these cohorts were not specifically designed to assess generational mental health issues or the pandemic’s long-term impacts.

The 2019 Rio Grande birth cohort was created to study the transmission of mental health traits and disorders across generations. The study also examines socioeconomic conditions, determinants of maternal and family health, access to and use of health services, mother-infant interactions, family characteristics, child development, and morbidities from birth onward.

## STUDY PARTICIPANTS

Rio Grande, a municipality in southern Brazil, has a population of 212,673 residents. In 2020, it ranked 698th in gross domestic product, 60th in average monthly income (Instituto Brasileiro de Geografia e Estatistica, 2020 and 2022), and 3533rd in the GINI coefficient (Brazilian national health system, 2010) [13]. The city records approximately 2,500 births per year and is unique in Brazil for conducting regular perinatal surveys every 3 years since 2007 [19]. These studies have evaluated prenatal care, perinatal health, and childbirth. With continued data collection, our aim is to investigate the health consequences for mothers and children in LMICs.

**Figure 1.**
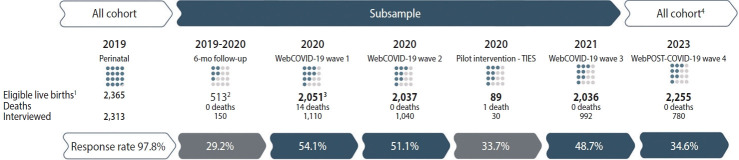
Flowchart of the 2019 Rio Grande birth cohort recruitment and follow-up. WebCOVID-19 wave 1: Complete questionnaires (>80%): 1,021; partially complete questionnaires (50-80%): 43; incomplete questionnaires (<50%): 46; WebCOVID-19 wave 2: Complete questionnaires (>80%): 997; partially complete questionnaires (50-80%): 41; incomplete questionnaires (<50%): 2; WebCOVID-19 wave 3: Complete questionnaires (>80%): 895; partially complete questionnaires (50-80%): 27; incomplete questionnaires (<50%): 0; WebPOST-COVID-19: Complete questionnaires (>80%): 736; partially complete questionnaires (50-80%): 22; incomplete questionnaires (<50%): 22. ^1^Stillborn children (n=15). ^2^It were selected 25%, randomly. ^3^Eligible sample: after WebCOVID-19 elegibility criteria was applied. ^4^Single child from urban and rural areas (twins were not included).

Figure 1 presents the participant flowchart, illustrating the sample size for each follow-up, eligibility criteria, as well as losses and refusals. In 2019, there were 2,365 births in Rio Grande, and we interviewed 2,313 of these mothers. In the same year, a smaller study involving 513 females was launched, but only 150 responses were obtained due to the onset of the COVID-19 lockdown in 2020. During the pandemic, we conducted 3 online follow-ups called WebCOVID-19 (waves 1 to 3). Between May 2020 and July 2020, we interviewed 1,110 mothers from a pool of 2,051 eligible participants (limited to mothers with singletons living in the urban area in 2019). This online survey was repeated between July 2020 and December 2020. During this period, we identified 14 child deaths, reducing the eligible sample to 2,037, from which we interviewed 1,040 mothers. In 2020, we also piloted an intervention study, which is described later in the text. In 2021, another online follow-up was conducted, identifying 1 additional child death, resulting in 2,036 eligible participants and 992 interviews. In 2023, we conducted a fourth follow-up, WebPOST-COVID, expanding eligibility to include mothers with twins and those residing in rural areas. Altogether, 2,255 participants were eligible, and 780 were interviewed.

### Perinatal (baseline)

This is a fixed, prospective birth cohort that includes mothers who gave birth between January 1, 2019 and December 31, 2019. All mothers of liveborn infants in 2019 at 1 of the 2 maternity hospitals in the city, whose infants weighed ≥500 g or had reached at least 20 weeks’ gestational age, were invited to participate. The hospitals—the University Hospital of the Federal University of Rio Grande (HU-FURG) and the Santa Casa de Misericórdia of Rio Grande (SCMRG)—account for 99.5% of births among residents of the municipality [19]. All eligible mother-child dyads were interviewed at the hospital within 48 hours after delivery.

### WebCOVID-19 (waves 1 to 3)

The WebCOVID-19 follow-up surveys were conducted online. Eligible participants included mothers with single pregnancies residing in the urban area of Rio Grande at the time of the perinatal study. We identified 2,051 eligible mothers and attempted to contact each by web or telephone, making at least 3 attempts at different times to invite participation in the first wave (May-July 2020).

The research team was trained to locate mothers through social networks, send questionnaire links, and administer the survey by phone if necessary. A pilot study was conducted with non-participant mothers from the Rio Grande cohort to test the questionnaire, distributing an electronic link via WhatsApp along with a password for identification.

The same methods were used in waves 2 and 3 of WebCOVID-19. The second wave took place between July 2020 and December 2020, and the third wave occurred between October 2021 and May 2022.

### WebPOST-COVID (wave 4)

From June 2023 to April 2024, we conducted a new follow-up called WebPOST-COVID. For this wave, we expanded our inclusion criteria to mothers living in both urban and rural areas of Rio Grande. In total, 2,255 individuals were eligible, and 780 participated in the follow-up.

### Sub-studies

#### Six-month follow-up

The first follow-up of the 2019 Rio Grande birth cohort, “Rio Grande Birth Cohort: A Study on Child Development and the Quality of Life of Families,” was conducted when the children were 6 months old. This study aimed to initiate longitudinal follow-up. We randomly selected 20% of the children born each month in 2019, yielding an estimated sample size of 513, with 25% from urban areas and single births. From August 2019 to February 2020, trained interviewers conducted face-to-face interviews with mothers at their homes. In February 2020, Brazil reported its first COVID-19 case, leading to the suspension of in-person interviews due to social distancing measures. Consequently, this follow-up included only 150 participants (29.2% of the estimated sample size).

#### TIES pilot intervention

We developed text-message intervention to enhance social support (TIES) to increase maternal social support during the COVID-19 pandemic and evaluated its acceptability and feasibility. The study included mothers from the 2 oldest age tertiles with complete 6-month assessment data. Of 89 eligible families, we randomly contacted 74 mothers and enrolled 30 participants. The intervention provided education and support for mothers of children aged 12-18 months over 8 weeks, focusing on mother-child health, interaction, and maternal well-being. Outcomes assessed included maternal mental health, child nutrition, sleep, and behavior, with emphasis on child nutrition, sleep, early learning, responsive caregiving, and maternal psychosocial health. Further details are available elsewhere [20].

### Ethics statement

The Rio Grande University Ethics Committee (health sector) approved this cohort under protocol No. 15724819.6.000.5324, and all participants provided informed consent. The study followed the principles outlined in the Declaration of Helsinki.

## MEASUREMENTS

We developed questionnaires for the baseline, follow-up waves, and sub-studies using REDCap software, which allows direct data entry during interviews [21]. This software, licensed from Vanderbilt University Medical Center, enables immediate data input, eliminates the need for typists, and accelerates the time between data collection and dataset readiness for analysis. Further details are available elsewhere [22]. Table 1 summarizes the variables collected during each follow-up or sub-study.

Mental health is a key measure in this cohort. The following section details the data collection instruments; more information is provided in Supplementary Material 1:

(1) Maternal stress due to the pandemic: We used the Brazilian version of the Impact of Event Scale (IES), which demonstrated high reliability in assessing maternal stress related to the pandemic. The instrument underwent forward and backward translation for cross-cultural validation and showed a sensitivity of 81.4%, specificity of 70.0%, and a Cronbach’s alpha of 0.93 [14]. The IES comprises 15 items with 4 response options (total score range 0-75), and a cut-off score of 26 was used to identify moderate to severe stress. The application time is approximately 6 minutes [23].

(2) Maternal depression: The Edinburgh Postnatal Depression Scale (EPDS) [17] was translated and validated for Brazilian populations, with a sensitivity of 59.5% and specificity of 88.4% using a cut-off of 13. This 10-item questionnaire takes about 5 minutes to complete and has a total score range of 0-30 [24].

(3) Maternal anxiety: The Generalized Anxiety Disorder-7 (GAD-7) tool has demonstrated cultural applicability and high internal consistency (composite reliability of 0.91) for assessing generalized anxiety symptoms in a Brazilian population. The GAD-7 consists of 7 items with 4 response options, for a total score range of 0-21; a cut-off of 10 was used to indicate moderate to severe anxiety. The average application time is about 2 minutes [25].

### Perinatal component (baseline)

The perinatal study focused on maternal health during pregnancy and immediately after delivery. It assessed prenatal care, pregnancy morbidities, use of folic acid and ferrous sulfate, back pain, hospital admissions, labor assistance, birth companion, pain management, child health and physical examination, breastfeeding, use of pacifiers and bottles, infant sleep position and location, vaccinations, oral health, reproductive history, smoking, alcohol use, physical activity, perceived social support, and maternal mental health, among other variables [19].

### WebCOVID-19 (waves 1 to 3)

The WebCOVID-19 follow-ups assessed the impact of the pandemic on socioeconomic conditions, maternal mental health, purchasing habits, time spent outside the home, and use of personal protective equipment such as masks. Data were collected on mothers’ general and mental health (depression, anxiety, stress), infants’ health, and mother-child interactions (Table 1).

The WebCOVID-19 follow-ups evaluated maternal mental health, child development, COVID-19 symptoms, vaccination status, healthcare access, coping strategies, domestic violence, maternal sleep, physical activity, social support, oral health, and Centers for Disease Control and Prevention child development milestones. Maternal lifestyle factors such as smoking, alcohol use, and parenting behaviors were also recorded.

### WebPOST-COVID (wave 4)

This wave focused primarily on child outcomes, including emotional symptoms, conduct problems, hyperactivity, and aggressive behaviors. We used the Brazilian version of the Strengths and Difficulties Questionnaire (SDQ) to assess child mental health, which includes 25 items divided into 5 subscales: emotional problems, hyperactivity, peer relationships, conduct problems, and prosocial behavior [26]. We also gathered data on oral health, maternal sexuality behaviors, and continued to monitor maternal mental health using the EPDS. Other domains included work division and sexual dissatisfaction.

### Next steps and projects

In May 2024, Rio Grande do Sul and Rio Grande in Southern Brazil experienced severe flooding, resulting in extensive damage. The region received the equivalent of 6 months of rainfall in under 15 days, affecting over 3 million hectares, displacing 2 million people, and causing 200 fatalities. To address the consequences of this disaster, we have initiated a new online follow-up in 2025, when the children will be 6 years old.

We plan to repeat selected questionnaires, collect new data, and recruit new cohort members not included in the previous follow-up. New domains will include flood-related outcomes, trauma, eco-anxiety, attitudes toward corporal punishment, and domestic chaos. Fathers will also be surveyed regarding mental health, parenting, attitudes toward corporal punishment, and sex-equitable norms. We also intend to link official registry data on participant mortality and cause of death, family crime records, and academic performance during the next year. Furthermore, we have been collecting data on participants’ residences and geolocating them for use as exposure or outcome variables in future studies.

We will conduct a pilot study using first-person-view cameras to record home interactions between mothers and children. This will help us explore links between parenting practices, mother-child interactions, and child mental health, and refine our coding system for parental behaviors.

In 2025, we will record the interactions of 100 mothers using this method. The video data will be analyzed using the Noldus Observer XT [27] and FaceReader modules for real-time emotional analysis. During structured play sessions, both mothers and children will wear headcams to record their interactions at home. These videos will be analyzed with artificial intelligence-powered facial recognition software to assess attention, facial expressions, vocalizations, and maternal criticism, with the aim of understanding the impact of flood displacement on mother-child interactions and mental well-being.

Finally, we are applying for new grants to support future in-person follow-ups and to continue our online initiatives over the next 5 years. These proposals are being developed in collaboration with international researchers and other cohort leaders to ensure the collection of reliable and diverse data, thereby strengthening the robustness of our findings.

## KEY FINDINGS

The response rates for the follow-ups were as follows: face-to-face 6-month: 29.2%, n=150; WebCOVID-19 waves 1, 2, and 3: 54.1%, n=1,110; 51.1%, n=1,040; 48.7%, n=992, respectively; and WebPOST-COVID: 34.6%, n=780 (Figure 1). For the TIES study, recruitment was stopped after enrolling 30 participants out of 89 eligible mothers, as this was the required sample size.

Table 2 presents the cohort’s socio-demographic characteristics across follow-ups (participant sex and maternal reported skin color) and provides a descriptive analysis of mental health variables at baseline and across all 2019 Rio Grande birth cohort follow-ups. The composition of the sample remained consistent, with approximately 51% males and 80% of mothers reporting white skin color in each assessment.

Although the overall follow-up rate was low, a comparison between the perinatal study and each follow-up, based on baseline characteristics, is presented in Supplementary Material 2. Apart from differences in child skin color, there were no statistically significant differences between individuals who participated in the follow-ups and the original cohort.

### Main results to date

Although this article presents a cohort profile to serve as a reference for future publications, some findings have already been published and others are under review. The following is a summary of the principal published findings:

There was a 6-fold increase in depression prevalence in WebCOVID-19 wave 3 compared with baseline (34.7 vs. 5.9%), followed by a decrease of 7 percentage points in WebPOST-COVID (27.7%). Additionally, anxiety prevalence tripled in WebCOVID-19 wave 3 relative to baseline, while stress levels remained stable in WebCOVID-19 waves 2 and 3, both above 42%. These results indicate a marked increase in maternal depression and anxiety during the pandemic; more than 1 in 4 mothers experienced either depression or anxiety, and nearly half had moderate or severe stress related to COVID-19 [28].

Birth cohort evidence reveals varying patterns of prenatal and postnatal mental health, though some consistencies exist. For example, a study in Turkey found prenatal depression at 14.6%, rising to 18.5% at 6 months postpartum, while pregnancy anxiety was 29.6% and declined over 6 months [29]. Depression typically peaks 4 weeks to 6 weeks postpartum and gradually decreases, but may persist up to 5 years [29-31]. Similarly, anxiety decreases from pregnancy to the postpartum period, though long-term trajectories may vary [29-31]. Most studies show postnatal depression rises 2-fold to 3-fold after delivery, while anxiety tends to decrease [29-31]. Studies from Brazil display a similar pattern, with the prevalence of depression at 24 months generally no more than double the baseline rate [32,33].

In our cohort, the increases in depression and anxiety exceeded expectations and predictions from both international and local studies, likely due to the pandemic. Mental health issues among mothers increased sharply during COVID-19, indicating an urgent need for targeted interventions. Young females were particularly affected, exposing vulnerabilities in maternal and child health systems [34,35]. Scaling up maternal mental health care interventions in LMICs remains a major challenge requiring urgent attention [36-40]. Regional differences should be considered and local evidence prioritized to improve screening and treatment. Our findings underscore the necessity of addressing maternal mental health during and after crises like the COVID-19 pandemic. Longitudinal studies, including this one, are crucial for evaluating and understanding the long-term effects on maternal and child mental health.

We observed an inverse association between the gender division of labor among couples and psychological and sexual intimate partner violence (IPV). The division of labor was a strong predictor of IPV during the pandemic [41]. Furthermore, mothers who expressed fear for their children’s health and future exhibited increased depressive and anxiety symptoms during the pandemic [42].

The results presented in submitted manuscripts reveal several significant findings: Maternal depression and anxiety increased during the pandemic, negatively impacting interactions with children, parenting practices, and child development. Higher maternal alcohol consumption was associated with increased risk of these mental health issues. Additionally, food insecurity disproportionately affected highly vulnerable mothers, further exacerbating existing inequalities during the pandemic.

## STRENGTHS AND WEAKNESSES

We experienced over 50% loss to follow-up in all waves, which is common in online and remote research settings. Online studies typically achieve lower response rates than traditional research designs [43]. Even large and well-established cohorts, such as the 2015 Pelotas birth cohort, reached only a 50% follow-up rate during their own web-based COVID follow-up study [44].

Given that selection bias could potentially affect our cohort, we used propensity score analysis and inverse probability weighting (IPW) to generate an index reflecting the probability of being followed, based on baseline socio-demographic characteristics. This variable was incorporated into our final analyses to provide appropriate p-values and 95% confidence intervals in our publications [42].

The propensity score analysis yields an index variable representing the likelihood of follow-up participation, determined by baseline socio-demographic factors. Logistic regression analyses were conducted using non-response during the pandemic as the outcome, with baseline covariates independently associated with non-response serving as predictors in multivariable models. Covariates included age, education, income, cohabitation status, and self-reported skin color. The model is updated for each analysis, as missing data in the outcome variable may vary depending on the follow-up wave or response rates. The fit of the missingness models and the distribution and stability of weights were evaluated using the Hosmer-Lemeshow test, which indicated a good fit (p>0.2). Predicted values from the final regressions were used to generate a weight variable with the propwt command in Stata using IPW. This variable represents the likelihood of follow-up participation based on baseline characteristics (age, education, income, living with a partner, self-reported skin color).

Selection bias arises when the probability of being included in the sample is not uniform and is related to both exposure and outcome, which can distort measures of association. In IPW analysis, a new variable is generated to account for each individual’s probability of inclusion, and this is used to weight the results. This method reduces the potential for bias, providing more accurate estimates of association, confidence intervals, and p-values in the final analyses.

A key strength of this study is the inclusion of a population-based prospective cohort from a middle-income country—a rarity in this region. While our web-based approach may yield lower follow-up rates, it enables data collection from participants who have left the Rio Grande area and from mothers with limited access to the research site. This approach is well-suited to a world increasingly reliant on technology and the internet. From a logistical perspective, it also facilitates the measurement of a limited set of key health indicators over short time periods.

By collecting data on births occurring before the pandemic and subsequently tracking the health of these children, we were able to examine the effects of COVID-19 on participants’ overall health. Our results indicate that the incidence of mental health problems among mothers has risen significantly since 2019. Although there was a slight decrease after the peak of the pandemic, both mothers and children continue to experience ongoing health effects [32]. Importantly, we have collected data using the same instruments over time, allowing for rigorous longitudinal analyses—albeit over shorter periods compared with other major regional cohorts [12,18].

Our study offers novel insights into maternal mental health, child development, and post-COVID-19 behaviors. This is a mental health-focused birth cohort, with intergenerational transmission of mental health as our primary research objective. We employ multiple methodologies, including innovative technologies, to examine culturally specific risk factors in the causal pathway of intergenerational mental health transmission.

## DATA ACCESSIBILITY

We invite collaborations for data analysis using the 2019 Rio Grande birth cohort data. Current collaborations include: (1) DOVE Research Center from the Federal University of Pelotas, Brazil; (2) Bristol Medical School from the University of Bristol, UK; (3) Department of Nutrition and Food Studies, New York University USA.

Researchers interested in building networks among different institutions are encouraged to contact us. To request access to our data for potential publications, please e-mail: coorteriogrande2019@gmail.com to obtain our data access form. Upon receiving the form, the researchers must develop a detailed project proposal that includes a justification for the request, clearly defined objectives, an analysis plan, and any proposed compensation issues.

As our data are not fully open-access to protect personal identities and due to the absence of participant consent, the cohort coordinators will evaluate proposals for further collaborations based on their feasibility and potential overlap with ongoing studies. If accepted, anonymized datasets and data dictionaries will be provided. The researchers will be encouraged to share any variables created and their corresponding codes after completing their analyses.

## Figures and Tables

**Table 1.** Instruments and subjects investigated at baseline (perinatal) and follow-ups of the 2019 Rio Grande birth cohort

Variables	Perinatal	WebCOVID-19			TIES intervention	WebPOST-COVID -19
		Wave 1	Wave 2	Wave 3		
Brief Infant Sleep Questionnaire			✓			
Breastfeeding	✓	✓	✓			
Child mental health						✓
Child development				✓		✓
Child food neophobia					✓	
COVID-19 symptoms		✓				
Diseases during pregnancy and prenatal	✓					
Division of labor			✓			
Edinburgh Postnatal Depression Scale	✓	✓	✓	✓	✓	✓
Food insecurity		✓				
Generalized Anxiety Disorder	✓	✓	✓	✓		
Home Observation for Measurement of the Environment					✓	
Impact of COVID-19 on families’ life		✓	✓	✓		
Impact of Event Scale		✓	✓	✓		
Intimate partner violence			✓	✓		
Healthcare needs and access		✓	✓			
Labor and newborn’s health	✓					
Maternal oral health^1^			✓			✓
Maternal health and behaviors	✓					✓
Maternal sexual satisfaction						✓
Maternal sleep	✓			✓		
Scale of Perceived Social Support					✓	
Obstetric violence	✓					
Parenting and Family Adjustment Scales			✓			
Protection measures against COVID-19		✓	✓	✓		
Religion	✓					
Reproductive history	✓					
Self-efficacy					✓	
Socio-demographic characteristics	✓	✓	✓	✓	✓	✓

Instruments measured during the 6-month follow-up are not shown due to the cancelation of this assessment as a result of the COVID-19 pandemic. TIES, text-message intervention to enhance social support; COVID-19, coronavirus disease 2019.

1 General oral health, bruxism, and temporomandibular disorders.

**Table 2.** Sample characteristics of the 2019 Rio Grande birth cohort waves

Characteristics	Perinatal (2019)	WebCOVID-19			WebPOST-COVID-19 Wave 4 (2023-24)
		Wave 1 (2020)	Wave 2 (2020)	Wave 3 (2021-22)	
Sex					
Male	1,181 (51.1)	563 (50.7)	540 (51.9)	480 (52.1)	408 (52.3)
Female	1,132 (48.9)	547 (49.3)	500 (48.1)	442 (47.9)	372 (47.7)
Skin color					
White	1,763 (76.3)	898 (80.9)	830 (79.8)	729 (79.2)	611 (78.6)
Non-white	546 (23.7)	212 (19.1)	210 (20.2)	191 (20.8)	166 (21.4)
Depression	134 (5.9)	309 (29.5)	293 (29.4)	313 (34.7)	206 (27.7)
Anxiety	246 (10.7)	268 (25.9)	297 (28.5)	303 (33.1)	-
Stress	-	438 (40.9)	416 (40.0)	393 (43.8)	-

Values are presented as number (%).

## Supplementary materials

Supplementary Material 1. Detailed information about the main outcomes used in the 2019 Rio Grande Birth Cohort study

Supplementary Material 2. Comparison between the perinatal study and each follow-up (WebCOVID-19 Waves 1-3 and WebPOST-COVID-19 studies) according to baseline characteristics
