# Supplementary material for: The 2019 Rio Grande birth cohort: profile of a Brazilian 5-year study on mental health conditions
Source: Epidemiol Health. 2025 Jul 21;47:e2025039. doi: 10.4178/epih.e2025039 (PMC12531462; doi:10.4178/epih.e2025039)
Supplement: Supplementary Material 1. — Detailed information about the main outcomes used in the 2019 Rio Grande Birth Cohort study [file epih-47-e2025039-Supplementary-1.docx]

Supplementary Material 1. Detailed information about the main outcomes used in the 2019 Rio Grande Birth Cohort study

|  | **IES** | **EPDS** | **GAD-7** |
| --- | --- | --- | --- |
| Sensitivity (%) | 81.4 | 59.5 | - |
| Specificity (%) | 70 | 88.4 | - |
| Cronbach’s α | 0.9 | 0.8 | 0.9 |
| Number of items | 15 | 10 | 7 |
| Range of score | 0-75 | 0-30 | 0-21 |
| Cutoff for the outcome | 26 | 10 | 10 |
| Time of application (minutes) | 6 | 5 | 2 |

*IES: Impact Event Scale; EPDS: Edinburgh Postnatal Depression Scale; GAD-7: Generalized Anxiety Disorder Questionnaire
