# Supplementary material for: The 2019 Rio Grande birth cohort: profile of a Brazilian 5-year study on mental health conditions
Source: Epidemiol Health. 2025 Jul 21;47:e2025039. doi: 10.4178/epih.e2025039 (PMC12531462; doi:10.4178/epih.e2025039)
Supplement: Supplementary Material 2. — Comparison between the perinatal study and each follow-up (WebCOVID-19 Waves 1-3 and WebPOST-COVID-19 studies) according to baseline characteristics [file epih-47-e2025039-Supplementary-2.docx]

**Supplementary Material 2. Comparison between the perinatal study and each follow-up (WebCOVID-19 Waves 1-3 and WebPOST-COVID-19 studies) according to baseline characteristics**

|  | **Perinatal**  **(2019)** | **WebCOVID-19 Wave 1**  **(2020)** | **WebCOVID-19 Wave 2**  **(2020)** | **WebCOVID-19 Wave 3**  **(2021-22)** | **WebPOST-COVID-19**  **(2023-24)** |
| --- | --- | --- | --- | --- | --- |
|  | **N (%)** | **N (%)** | **N (%)** | **N (%)** | **N (%)** |
| **Sex** |  | p=0.771 | p=0.477 | p=0.445 | p=0.403 |
| Male | 1181 (51.1) | 563 (50.7) | 540 (51.9) | 480 (52.1) | 408 (52.3) |
| Female | 1132 (48.9) | 547 (49.3) | 500 (48.1) | 442 (47.9) | 372 (47.7) |
| **Skin color** |  | **p<0.001** | **p<0.001** | **p=0.008** | p=0.070 |
| White | 1763 (76.3) | 899 (81.0) | 830 (79.8) | 729 (79.2) | 611 (78.6) |
| Non-white | 546 (23.7) | 211 (19.0) | 210 (20.2) | 191 (20.8) | 166 (21.4) |
| **Depression** |  | p=0.109 | **p=0.004** | p=0.467 | p=0.157 |
| No | 2162 (94.1) | 1048 (95.0) | 989 (95.7) | 866 (94.6) | 733 (95.2) |
| Yes | 134 (5.9) | 55 (5.0) | 44 (4.3) | 49 (5.4) | 37 (4.8) |
| **Anxiety** |  | p=0.225 | p=0.119 | p=0.130 | ^Ω^ |
| No | 2061 (89.3) | 999 (90.2) | 939 (90.5) | 809 (88.1) | 677 (87.3) |
| Yes | 246 (10.7) | 109 (9.8) | 99 (9.5) | 109 (11.9) | 98 (12.7) |
| **Stress** |  | ^¥^ | ^¥^ | ^¥^ | ^¥^ |
| No | ^¥^ | ^¥^ | ^¥^ | ^¥^ | ^¥^ |
| Yes | ^¥^ | ^¥^ | ^¥^ | ^¥^ | ^¥^ |

*p-values calculated from Fisher exact test comparing participants from each follow-up with the baseline participants according to the baseline variables

^¥^Impossible to estimate p-values as we did not apply the stress questionnaire (IES) in the perinatal follow-up

^Ω^Impossible to estimate p-value as we did not apply the anxiety questionnaire (GAD-7) in the WebPOST-COVID-19 follow-up
